# CCUS development in China and forecast its contribution to emission reduction

**DOI:** 10.1038/s41598-023-44893-y

**Published:** 2023-10-19

**Authors:** Pengchen Wang, Beibei Shi, Nan Li, Rong Kang, Yan Li, Guiwen Wang, Long Yang

**Affiliations:** 1 School of Economics and Management, Northwest University, Xuefu Avenue No.1, Chang’an District, Xi’an, 710127 China; 2 China Energy JinJie Energy Co., Ltd, JinJie Industrial Park, Shenmu, 719319 Yulin China

**Keywords:** Projection and prediction, Environmental impact

## Abstract

Nowadays environmental issues have been of great concern to the world, among which the problem of global warming caused by greenhouse gas emissions is particularly prominent. All countries in the Kyoto Protocol and the Paris Agreement have committed to control greenhouse gas emissions, and China, as the largest carbon emitter, has assumed a heavier burden. China has been striving to develop low-carbon technologies such as hydrogen, nuclear, wind, and solar energy, but the most attention should be paid to CCUS, which many scholars have high expectations that CCUS can help China reduce emissions to some extent. Therefore, this paper presents a prediction that CCUS can reduce 3.8% of carbon emissions for China in 2040 when CCUS emission reductions increase at a rate of 30%. The power and chemical industries could reduce carbon emissions by 2.3% and 17.3%, respectively.

## Introduction

Since industrialization, human activities have caused global temperatures to rise by about 1 °C, and if global warming continues at its current rate, temperatures will rise by 1.5 °C between 2030 and 2052. In turn, greenhouse gas emissions are one of the main causes of warming, with carbon dioxide emissions accounting for the majority of greenhouse gas emissions^1^. In the Paris Agreement, countries have pledged to address this environmental issue by proposing their own climate solutions. As of April 2021, 44 countries and the EU have announced net-zero emissions targets, and these countries and regions have pledged to reduce emissions by 70% of global ${\text{CO}}_{2}$ emissions.

In order to reduce the increasing concentration of ${\text{CO}}_{2}$ In the atmosphere, countries have made many efforts in the last few decades to reduce the consumption of fossil energy, to develop renewable energy sources such as wind, nuclear and hydrogen, to use fuels with shorter carbon chains and CO_2_ capture and storage technologies, etc^2^. In particular, carbon dioxide capture and storage (CCS) is considered to make a significant contribution to global emissions reduction by being used in conjunction with a number of emission reduction options^3^. CCS technology could reduce global emissions by 50–85% by 2050^4^.

China's resource endowment determines the country's "coal-rich, oil-poor, and gas-poor" energy mix, making most of China's CO_2_ emissions come from fossil fuel combustion^5^. In 2012, 68% of the country's CO_2_ emissions came from burning coal, with oil accounting for about 13% and natural gas for about 7%. As the world's largest emitter of CO_2_, China's economy is highly dependent on fossil energy sources, and the advent of CCUS technology can greatly mitigate the impact on China's economy when dealing with climate issues. The first CCUS project ran smoothly in 2005, and as the country's attention to climate issues has grown, CCUS technology has gained significant momentum in China, with about 40 projects currently in operation or running intermittently. In September 2020, China proposed a "double carbon goal" of achieving peak carbon by 2030 and achieving carbon neutrality by 2060. Compared to developed countries, China has only 30 years to reach peak carbon and become carbon neutral. As an important technology in the field of carbon emission reduction, CCUS is crucial to China's emission reduction. According to relevant research institutions, under the carbon neutrality target, China's CCUS emission reduction demand is 20- 408 million tons in 2030 and 0.6–1.45 billion tons in 2050. However, after fifteen years of development from 2005 to 2020, the total emission reduction from operating CCUS projects is only 3.298 million tons. Most scholars have focused on CCUS emission reductions by 2040 or 2060 but have neglected the development process of how to achieve these desired reductions.

This paper sets three development rates, high, medium, and low, to obtain the emission reduction contribution of CCUS at the year 2040. The emission reduction contribution of CCUS is obtained from another perspective and compared with the expected value to consider what growth rate we use to develop CCUS technology is the most appropriate and beneficial for China's economy.

## Literature review

### Review of CCUS-related literature

The concept of CCUS evolved from CCS, from the initial single storage of CO_2_ to utilization and storage. Domestic and international research on CCUS has focused mainly on CCUS deployment, CCUS economic value and CCUS reduction potential etc. Vishal et al.^6^ combine CCUS with advanced technologies in India to study the strategy of CCUS technology promotion in India while considering the differences in carbon utilization in industries with different carbon intensity. Bazhenov et al.^7^ evaluated CCUS in the context of the Carbon Border Adjustment Mechanism (CBAM) proposed by the European Union and estimate the potential of CCUS to provide 270 MTPA emission reduction for the Russian industrial sector. Chen et al.^8^, based on a modeling analysis of the deployment of CCUS in China, concluded that the period 2040–2060 is the golden period for the development of CCUS in China. India, Russia, and China are all large CO_2_ emitters in the world, and the study of CCUS deployment pathways in each country proves that CCUS is already a key technology for net-zero emissions in each country, but all these studies only analyze the risk and cost issues in the deployment process, and they do not consider how the speed and slowness of the deployment will affect carbon neutrality.There are successful projects and there are also failed projects. Wang et al.^9^ estimated the risk of 263 CCUS projects and concluded that the risk of failure increases by about 50% when the emission reductions rises by every 1MT.To avoid the failure of the CCUS program, Storrs et al.^10^ analyzed 22 papers using meta-review to analyze the many challenges of CCUS development in terms of economic, technological, social, institutional, and organizational feasibility.

The study of the economic value of ccus includes two aspects. First, CCUS economic value studies focus on the cost differences and cost estimates for coal-fired plants and plants utilizing other capture technologies with and without CCUS under different exogenous variables. Rubin et al.^11^ compared the cost of pulverized coal plants (PC), natural gas combined cycle plants (NGCC), and integrated gasification combined cycle plants (IGCC) under the effects of natural gas price increases, plant utilization differences, IGCC financing and operating assumptions, and integrated gasification combined cycle power plants (IGCC) to compare the levelized cost of three types of power plants. From the perspective of levelized cost, Fan et al.^12^ compared the full-chain CCS projects of coal-fired power plants with various other low-carbon power generation technology plants and concluded that the full-chain CCS projects of coal-fired power plants have cost advantages but are influenced by coal prices and transportation distances, which shows that the carbon capture cost is lower after the CCUS retrofit to coal-fired power plants. Han et al.^13^ used a multidimensional benefit measurement system constructed by neural network algorithms and machine learning to study the various benefits of CCUS retrofitting in coal power plants, among which the environmental benefits were not focused on in previous studies, and the environmental benefits were much greater than the economic and energy benefits, while ccus projects take an average of 21.3 years to pay back their costs.

Second, in terms of investment decision, the study of CCUS investment decision mainly discusses the optimal timing of CCS investment under uncertain energy prices and policy factors; the investment scale of CCS is huge and has certain irreversibility, so the study of its optimal timing is crucial. First, considering uncertain energy prices, Fuss et al.^14^ analyzes the uncertainty of electricity and carbon prices as well as the policy and market uncertainties on this basis, and uses a real options model to derive the optimal timing for investing in CCS projects. Abadie and Chamorro^15^, on the other hand, consider the uncertainty of European electricity prices and carbon emission markets, and go further by using the European emission market to calibrate the emission rationing process. Oda^16^ uses a discounted cash flow approach to compare the break-even between rebuilding environmentally friendly power plants and retrofitting old coal plants to obtain the energy price for the optimal timing of CCS investment under the uncertainty of both carbon price and natural gas price. Second, when considering policy uncertainty, the above-mentioned scholars' study concluded that climate policy uncertainty would affect the promotion and utilization of CCS technology to some extent. Greig and Uden^17^ propose that threshold value, commercial value, and choice value together determine the role of CCUS in net-zero emissions, and that it is profitable to develop a CCUS when it has all three values.

### Review of literature in the field of carbon emissions

Research by scholars in the field of carbon emissions focuses on the prediction of carbon emissions, including both the prediction of carbon peaks and the prediction of carbon reduction potential.

Firstly, the main methods utilized in the research of carbon peaking prediction are grey models, ARIMA models, and STIRPAT models. Wang^18^ improves the traditional grey model by using a nonlinear multivariate grey model to predict carbon emissions from fossil energy consumption in China at different rates of economic development. The AIRMA model can better incorporate time related information, Sen et al.^19^ used ARIMA model to predict energy consumption and GHG emissions from pig iron manufacturing industry in India. Mohamed and Bodger^20^ used ARIMA model to include economic and demographic variables to predict electricity consumption in New Zealand. The prediction studies done by the above scholars are only for a single industry in a single country for energy consumption The forecasting studies done by the above scholars were only for a single industry in a single country. In contrast, the STIRPAT model is capable of integrated forecasting and is gradually becoming the most mainstream and widely used method in predicting carbon emissions models. Xu et al.^21^ utilized both the STIRPAT model and the GREY (1, 1) model to first classify total energy consumption into five types, and use the STIRPAT model to forecast each energy consumption, and use the gray model to predict economic growth, industrial structure changes, and energy structure changes, and combine them to get the prediction results of carbon emissions. Wang et al.^22^ used the STIRPAT model to screen the factors affecting China's carbon emissions, used these factors as input factors, and used the whale algorithm to optimize the parameters of the extreme learning machine to predict China's carbon emission data.

Second, the prediction of carbon emission reduction potential. Carbon emission reduction is an important link to achieve the double carbon goal, so the prediction of carbon emission reduction potential has gradually received the attention of many scholars. Guo^23^ selected China's industrial sector as the research object, adopted the economic accounting method, considered both structural and intensity emission reduction perspectives, and concluded that China's industry has great emission reduction potential. Zhao et al.^24^ evaluated the reduction potential and economics of CCUS for China's carbon emissions in 2060, and concluded that about 5×${10}^{8}$ t CO_2_ emission is reduced by capture utilization, and the whole-process cost is about − 1400 to 200 RMB/t; about 22×${10}^{8}$ t CO_2_ emission is reduced by capture storage, and the whole-process cost is about 200–450 RMB/t. This study, which is more of a static study, describes the emission reduction contribution of CCUS in China in 2060.

The above literature has explored the cost, investment, and business model studies of CCUS at the economic level, and the carbon peak projections and carbon reduction potential projections of carbon emissions at the forecasting level. Therefore, the previous literature mainly starts from CCUS and carbon emission itself and does not study the contribution of CCUS development to carbon emission reduction. Therefore, the marginal contribution of this paper is to predict the development of CCUS projects from the national level and to obtain the emission reduction contribution of CCUS projects to the whole country. The contribution of CCUS technology to emission reduction in different industries can be obtained by dividing the types of industries captured according to industries, and the contribution can be predicted for some industries with difficulties in decarbonization. By dividing countries into regions, we can get the future development of CCUS technology in different regions under different economic conditions and source-sink matching.

## CCUS development status and trend in China

As global climate change becomes more severe, countries agreed in the Paris Agreement to take measures to limit global temperature rise to 2 °C. Under this consensus, all countries have taken large-scale actions to reduce emissions, develop clean energy, adjust industrial structure, etc. However, a key technology, CCS, was proposed in the IPCC special report "Carbon Dioxide Capture and Storage" in 2004 and was listed as one of the key technical means to reduce emissions in the Kyoto Protocol in 2007, followed by the International Energy Agency (IEA), which also pointed out the importance of developing CCUS projects to reduce carbon emissions. According to the International Energy Agency (IEA) report "The Role of CCUS in Low-Carbon Power Generation Systems" published in 2020, electricity remains the most carbon-emitting sector of the energy industry, and fossil fuel combustion remains the largest source of electricity globally. Countries have already reduced carbon emissions from coal-fired units by 40% but will still emit 600,000 tons/year by 2040. Therefore, without large-scale application of CCUS technology, all coal and natural gas-fired power generation must be eliminated to achieve long-term global climate goals. In 2006, China first proposed the idea of carbon capture, utilization, and storage (CCUS), pointing out the importance of promoting CCUS technology for carbon reduction. The CCUS technology has been highly valued by the state and government since it was first proposed. The Chinese government has supported the basic research, technological breakthroughs and commercial operations of CCUS through the National Natural Science Foundation of China, the National Key Basic Research and Development Program ("973" Program), the National High Technology Research and Development Program ("863 Program"), the National Science and Technology Support Program and the National Key Research and Development Program. By the end of the 11th Five-Year Plan, there were more than 40 state-funded studies related to CCUS projects.

The development stages of CCUS in China can be roughly divided into four : CCUS was initially developed in China before 2006, when the China Union Coal ECBM project in Qinshui, Shanxi became the first project in China to utilize CO_2_;From 2006 to 2010, the development accelerated with an average of 1 new CCUS project per year and began to use CO_2_ drive to improve oil recovery and increase revenue for CCUS projects, such as the CNPC Jilin Oilfield EOR project and the CNPC East China Oil and Gas Field CCUS Full Process Demonstration Project. Although some new projects have been added, the overall emission reduction is still small, concentrated between 0.1 million tons–50,000 tons, and the demonstration significance of promoting the development of leading CCUS technology is much stronger. From 2010 to 2016, the development of CCUS has become more diversified, with a larger capture scale and new projects involving some tough emission reduction industries such as cement and chemical industries. For example, the Beijing Lulihe cement kiln tail flue gas carbon capture and application project is the first CCUS project in the cement industry. At this stage, 3–4 new CCUS projects are added every year, and the scale is also expanded compared with the previous stage, gradually expanding to a scale of 100,000 tons or more, such as the scale of Sinopec Zhongyuan Oilfield CO_2_-EOR project, which captures about 100,000 tons of CO_2_ per year. And after 2016, China's CCUS projects have developed even more rapidly, with some projects already capturing more than 1 million tons of CO_2_ per year.

### CCUS development and current status in China

#### CCUS project emission reduction status

**Figure 1:**
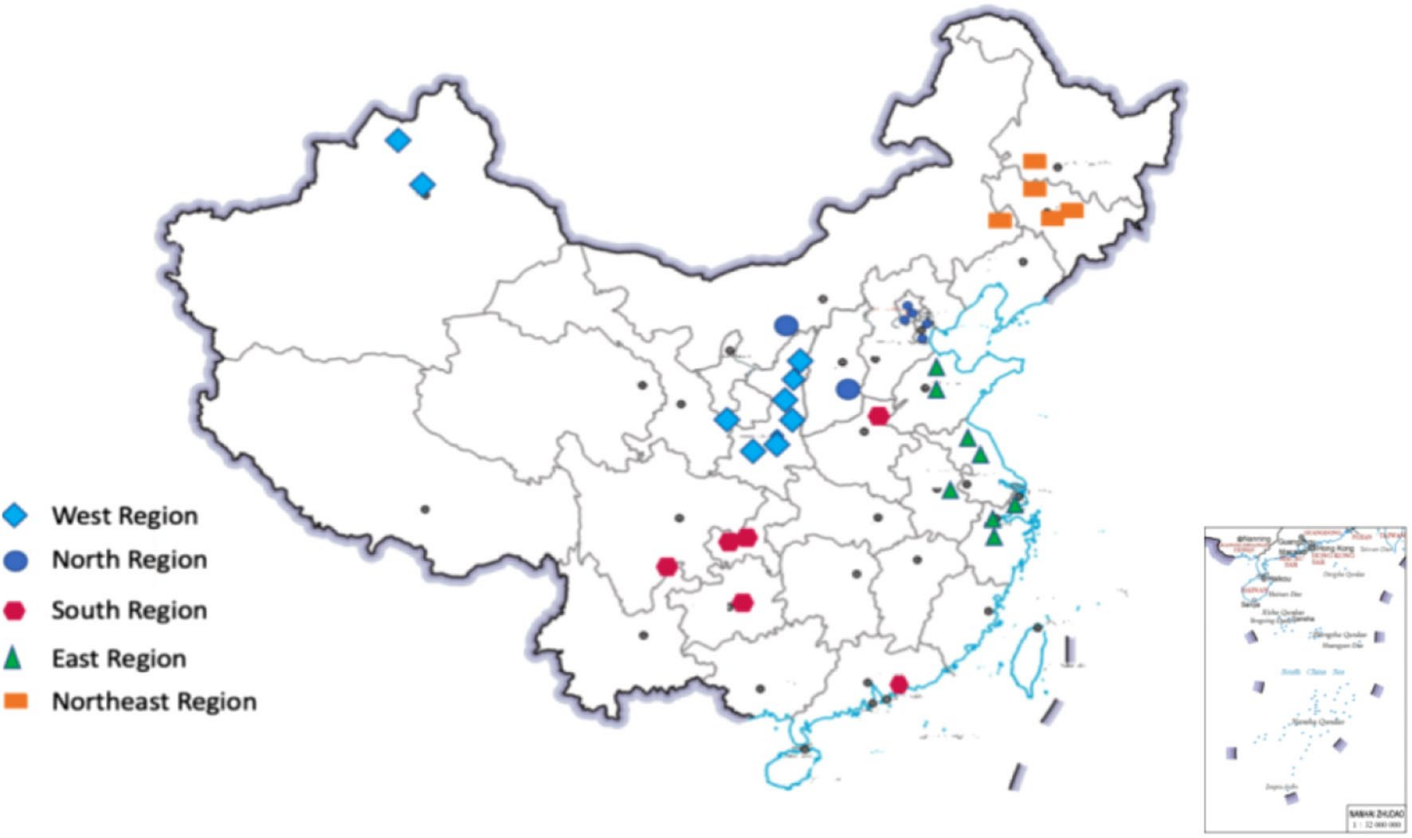
CCUS information location distribution map^25,26^.

**Figure 2:**
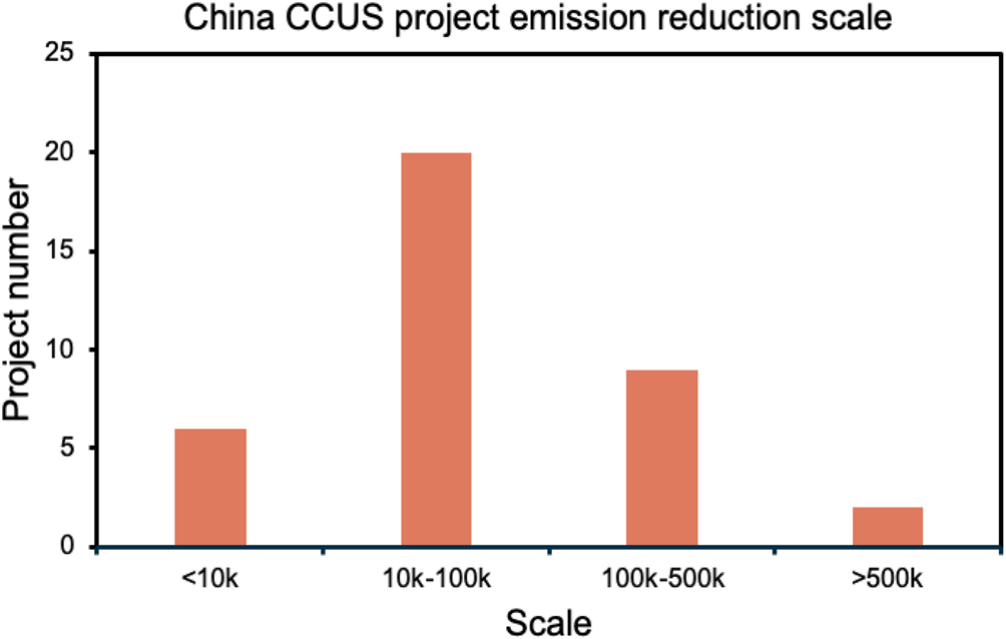
China CCUS project emission reduction scale.

At this stage, there are about 40 CCUS projects in operation in China, with a CO_2_ emission reduction of about 4 million tons/year. At present, China's CCUS projects are still mainly point-to-point projects, and no cluster projects have been formed. Most of the CCUS projects in operation are small-scale capture demonstration projects with emission reduction scales of 10,000 tons/year to 100,000 tons/year, and there are only two projects with emission reduction scales of more than 500,000 tons/year.The industries of CCUS projects in operation are mainly concentrated in the electric power, chemical industry as well as the iron and steel, and cement industries, with the chemical industry and the electric power industry accounting for more than 40% of the total. Geographically, CCUS projects are mainly concentrated in the eastern, western and northeastern regions. the location of projects is shown in the map in Fig. 1.The CCUS projects currently developed in China show a diversity of emission reductions, the regions where they are located, the types of industries captured, and the final utilization methods. The scale of emission reductions from projects ranges from 0.1 million tons to 1 million tons, with a major concentration between 10,000 tons and 500,000 tons which can be clearly seen from Fig. 2.

**Figure 3:**
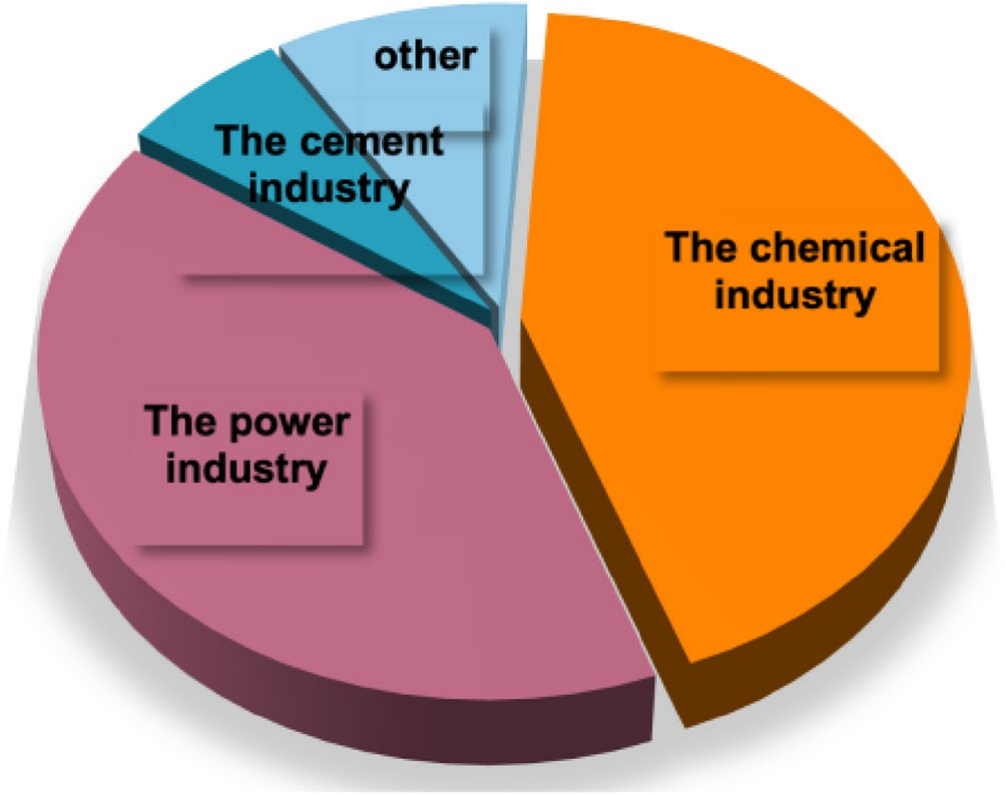
Distribution of catching industry types.

The types of industries captured include power, cement, and chemical industries as Fig. 3. shows the power industry in China is characterized by the structure of energy consumption, and China's power generation is dominated by thermal power, with coal-fired power generation accounting for 63% of the power industry^27^, and because of the high CO_2_ concentration and the less technical difficulty of retrofitting CCUS projects in thermal power plants compared to other industries, the power industry is the main industry where CCUS projects are applied. Secondly, the capture is concentrated in the tail gas emissions from cement plants and in the tail gas from the chemical industry for the preparation of chemical products. By 2021, up to 2.05 million tons of CO_2_ will be captured by the chemical industry.

#### Current status of CO_2_ utilization of CCUS project

**Figure 4:**
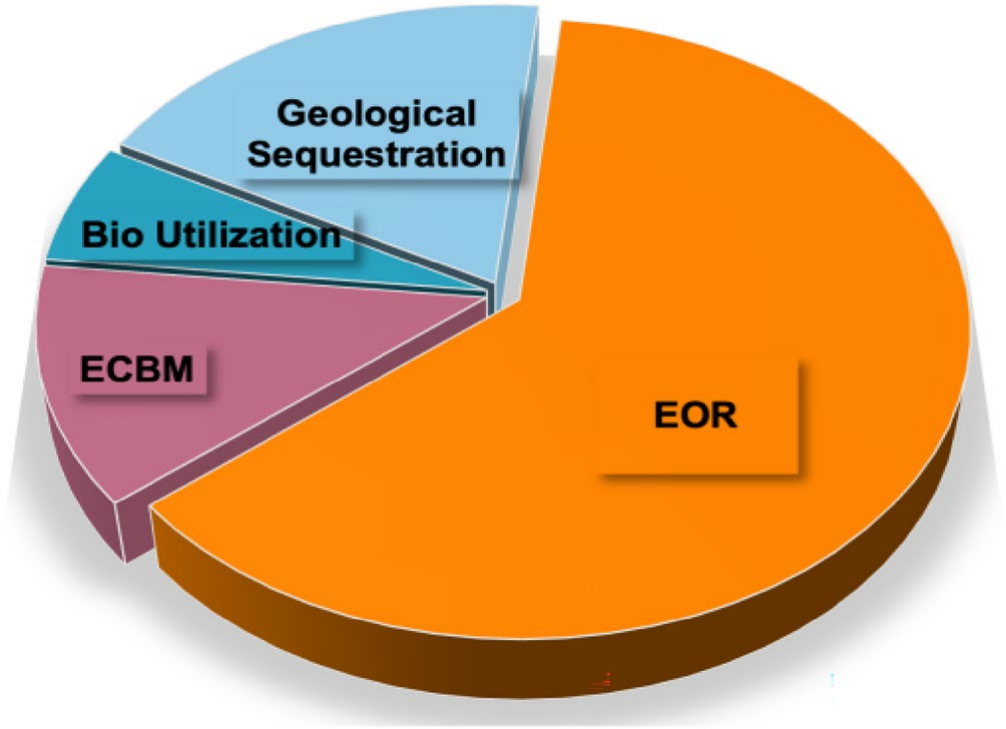
Distribution of captured CO_2_ utilization.

Final disposal of CO_2_ captured by CCUS includes Enhanced Oil Recovery (EOR), Enhanced Coal Bed Methane Recovery (ECBM), food grade CO_2_ refining, and other industrial uses.It can be seen from Fig. 4 that EOR is by far the most utilized method of CO_2_ and can increase oil recovery by 8–15% using CO_2_ drive^28^. The additional revenue ensures the stable operation of CCUS projects. The other approaches are subject to CO_2_ price fluctuations and sustainability of utilization methods, which bring relatively unstable benefits. Secondly, geological storage is also a common way to deal with CO_2_ in CCUS projects, but this method is limited by the distance between the carbon source and the storage site, economics, etc., and only 19% of projects currently use this method.

#### Current regional distribution of CCUS projects

**Figure 5:**
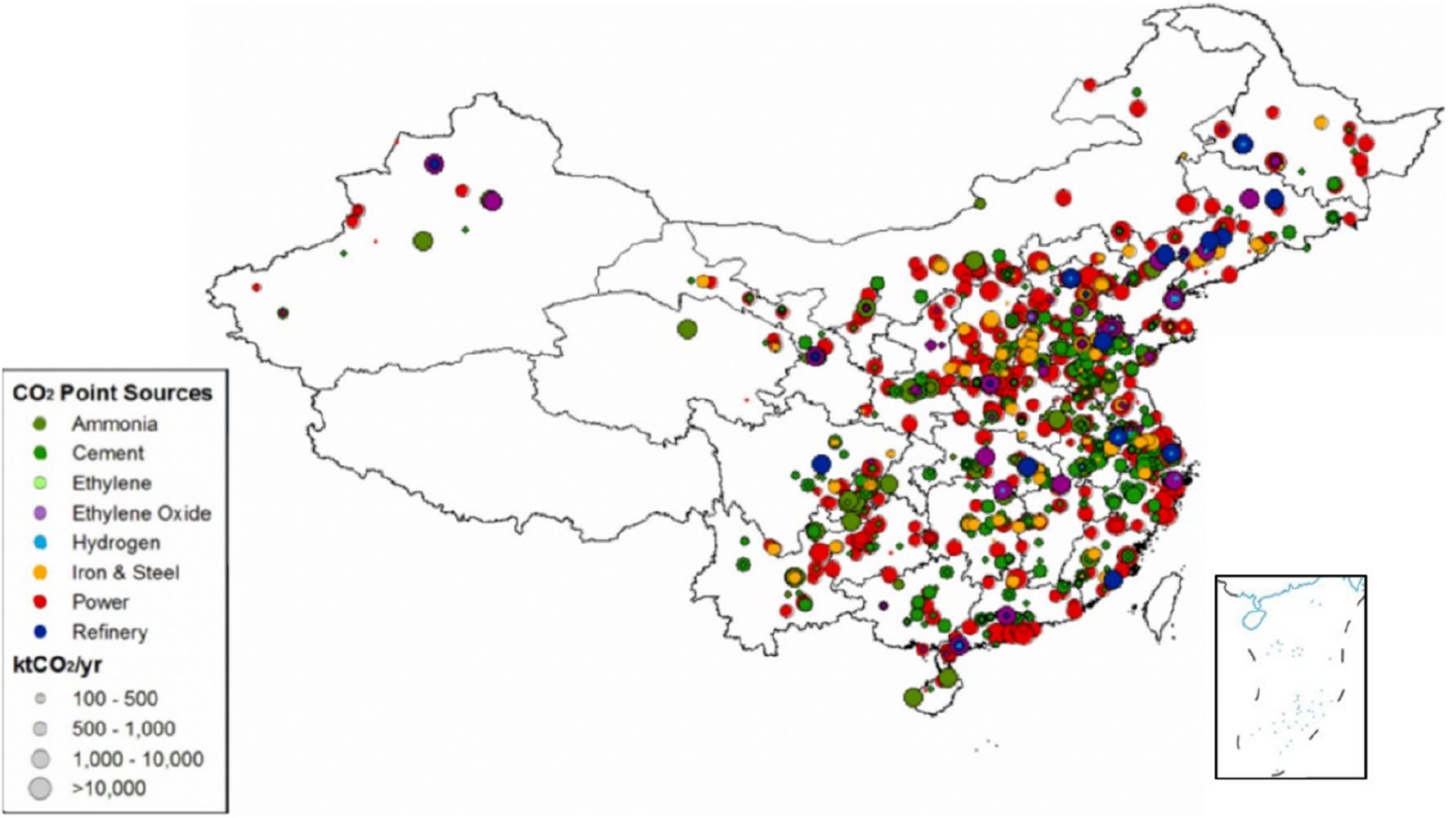
Distribution of large CO_2_ point sources in China^29^.

The distribution of CCUS projects is limited by the source-sink matching problem. On the one hand, CCUS projects need to have a stable and concentrated source of CO_2_ emissions, and on the other hand, they also need to have geological conditions that meet the requirements for CO_2_ storage or projects that can use CO_2_ to gain revenue. The distance between the storage site and the CO_2_ emission source should be within 250 km, because when the distance exceeds 250 km, CO_2_ pipeline transportation will require the construction of a transfer compressor station, which will significantly increase the cost of the project. Therefore, areas that meet the conditions for storage utilization and carbon sources will be more suitable for CCUS projects. Li et.al.^29^ estimated that there are about 1620 large carbon dioxide point sources in China, and their distribution is shown in the Fig. 5^29^.

In addition, CO_2_ disposal in CCUS projects in China is mainly through CO_2_-EOR drive and geological storage. From the perspective of basin storage potential and CO_2_ emission source distribution, the key areas where CO_2_-EOR can be implemented in China are the Songliao Basin region in northeast China, the Bohai Bay Basin region in north China, the Ordos Basin region in central China, and the Zhunger Basin region and the Tarim Basin region in northwest China. These are all onshore geologic sequestration locations and according to the calculation of Wei et al.^30^, the potential of onshore geological storage in China is about 2420 gt. Further, analyzing from county level, about 561 counties in China have geological storage capacity, and 40.6% of the counties have storage potential of more than 500 MT. From the location of large-scale CO_2_ emission sources and storage locations in China, it can be seen that although China has sufficient storage potential, there is a certain spatial mismatch in the source-sink. The eastern region, which has a large number of emission sources, does not have many geologic conditions suitable for storage. Faced with the problem of long distances between emission sources and storage locations, the cost of transportation by tanker trucks or trains may increase exponentially and bring more unpredictable risks, so the use of pipeline transportation needs to be considered.

### CCUS development trend in China

#### Impediments to the development of CCUS

The CCUS projects currently in operation in China are still in the industrial demonstration stage, and the demonstration projects are small in scale, with individual capture volumes mainly concentrated in the range of 1–500,000 tons. There are two main constraints to the development of CCUS projects in China; on the one hand, the cost for companies to operate the projects is too high. Additional capital investment and operating costs are required to build CCUS projects; for example, the cost of post-combustion capture projects in low-concentration coal-fired power plants, which belong to Huaneng Group, is 300 RMB/ton. When the Huaneng Group Shanghai Shidongkou Capture Demonstration Project was in operation, the cost of electricity generation rose from RMB 0.26 per kilowatt hour to RMB 0.5 per kilowatt hour^31^.On the other hand, the benefits of the project are unstable. While enhanced oil recovery through CO_2_ drive can bring significant benefits to CCUS projects, it is weaker in equally critical areas such as bio-utilization and chemical utilization. Chemical utilization currently generates only 1/6th of the revenue from address utilization. No stable revenue to generate positive cash flow for the business, making more than 80% of the projects unsustainable^25^.

#### CCUS future development direction

In order to cope with the high cost and energy consumption of CCUS projects, it is necessary to develop CCUS projects into full-scale demonstration projects, integrate existing resources and build CCUS industrial parks to reduce CO_2_ transportation and project operation costs. According to McKinsey's projections, when CCUS projects get past the initial demonstration phase, costs are expected to decrease by 10–20% for each doubling of the project size. Currently, new CCUS projects are being built with capture sizes of 500,000 tons or more, the basics of which are shown in Table 1 (project information are adopted from^25,26^).

**Table 1:** Projects currently under construction or proposed.

Project name	Location	Scale (MTPA)	Stage
Qilu Petrochemical Shengli Oilfield	Shandong	1	Advanced development
Tongyuan Oil Million Ton CO_2_ Capture and Utilization Integrated Demonstration Project	Xinjiang	1	Early development
China National Offshore Oil CCUS Project	Guangdong	1	Early development
CNPC 3 million tons CCUS project	–	3	Early development
Ningdong CCUS Demonstration Project Base	Ningxia	1	Early development
Longdong Carbon Capture Utilization and Storage Research and Demonstration Project	Gansu	1.5	Early development
Yanchang Oil CCUS Project	Shaanxi	5	Early development
Guanghui Energy CCUS Project	Xinjiang	3	Advanced development
National Energy Group carbon emission reduction utilization technology research and demonstration project	Jiangsu	0.5	Early development
Huating CO_2_ capture and utilization project	Gansu	0.5	Advanced development
Yulin Shenghui Hengtong CCUS project	Shaanxi	0.5	Advanced development

## Forecast CCUS contribution to emission reduction in China

This paper analyzes and predicts the potential of CCUS technology to contribute to emission reduction in China based on the aggregated data of emission reduction from operating or intermittently operating CCUS projects in China, combined with historical carbon emission data and predicted carbon emission data from some scholars.

### Forecast contribution to national emission reduction

By the end of 2020, China's CCUS emission reduction is 3.298 million tons. The emission reduction potential of CCUS is projected in two growth ways: one is the emission reduction potential under the growth of the number of CCUS projects, and the other way is the emission reduction potential under the growth of CCUS emission reduction.

#### Contribution under the growing number of CCUS projects

Referring to the development history of CCUS projects in China, in the early stages (2006–2010), one new CCUS project was added each year, while in 2010–2016, the state and government increased their attention and increased funding for research projects related to CCUS program development accelerated during this period, allowing for an average of 3 new projects per year. According to Table 1, it can be concluded that 70% of the CCUS projects that have been proposed or established are above 1 million tons of emission reduction, and large-scale projects are the development trend of future CCUS projects, thus setting the amount of emission reduction for new projects in the future at 1 million tons. Combined with the pace of China's energy consumption restructuring and the 2030 carbon peak target, the CCUS project should grow faster than the previous phase, or at least maintain the growth rate of the previous phase. Therefore, based on the past development rate, we set the number of new CCUS projects in each year in the future as ${\mathrm{k}}_{1}$=1, 2, 3, Forecast contribution of emission reductions from CCUS projects by 2040.

The emission reduction contribution can be calculated by the following equation:

$${\mathrm{r}}_{\mathrm{t}}=\left[\sum {\mathrm{CCUS}}_{{\mathrm{cap}}_{\mathrm{t}-1}}+{\mathrm{k}}_{1}*{\mathrm{CCUS}}_{{\mathrm{cap}}_{2020}}\times (\mathrm{t}-2020)\right]/{\mathrm{emis}}_{\mathrm{t}}$$

where, ${\mathrm{r}}_{\mathrm{t}}$ represents the emission reduction contribution of CCUS in year t, $\sum {\mathrm{ccus}}_{{\mathrm{cap}}_{\mathrm{t}-1}}$ is the cumulative emission reduction of national CCUS projects in year t−1, ${\mathrm{ccus}}_{{\mathrm{cap}}_{2020}}$ represents the emission reduction of CCUS in 2020, ${\mathrm{k}}_{1}$ represents the number of new projects each year, ${\mathrm{emis}}_{\mathrm{t}}$ represents the total national CO_2_ emission in year t, and $\mathrm{t}$ represents the year.

**Figure 6:**
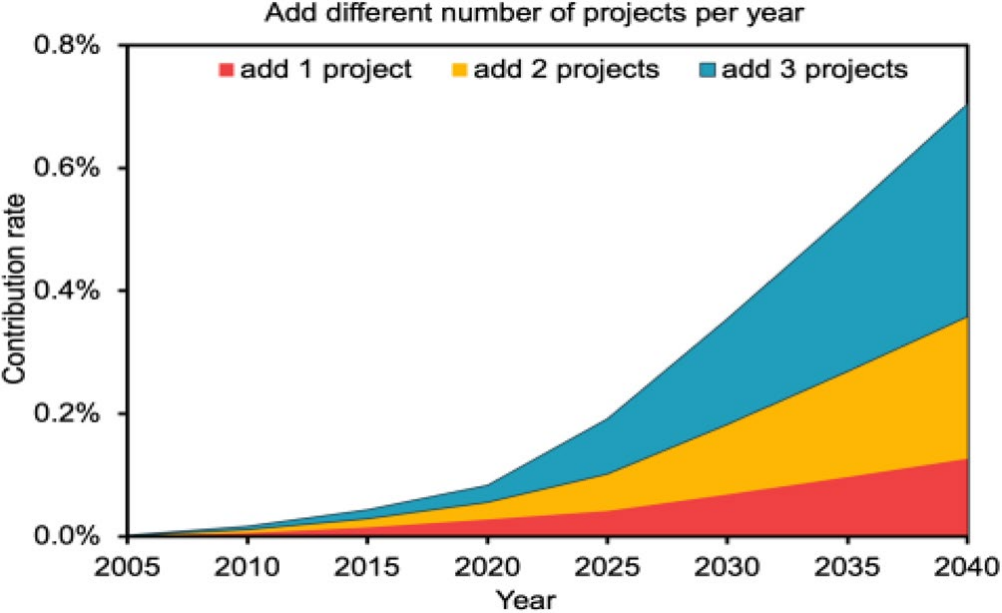
Add different number of projects per year.

From Fig. 6. we can see that when three new megaton CCUS projects are added each year, the national CO_2_ emissions in 2040 will be about 17,269 million tons, and the amount of CO_2_ captured by CCUS projects will be about 76,798,000 tons, and the contribution of CCUS technology will be about 0.34%.

#### Contribution under the growth of CCUS emission reductions

At the end of 2020, the total amount of carbon dioxide captured by the CCUS project is 3.298 million tons, and the annual growth rate of CCUS project emission reduction is set at ${\mathrm{k}}_{2}$, ${\mathrm{k}}_{2}$ takes 10%, 20%, 30% respectively ,to obtain the emission reduction contribution rate:

$${\mathrm{r}}_{\mathrm{t}}=\left[\sum {\mathrm{CCUS}}_{{\mathrm{cap}}_{\mathrm{t}-1}}+{\mathrm{CCUS}}_{{\mathrm{cap}}_{2020}}\times {(1+{\mathrm{k}}_{2})}^{(\mathrm{t}-2020)}\right]/{\mathrm{emis}}_{\mathrm{t}}$$

**Figure 7:**
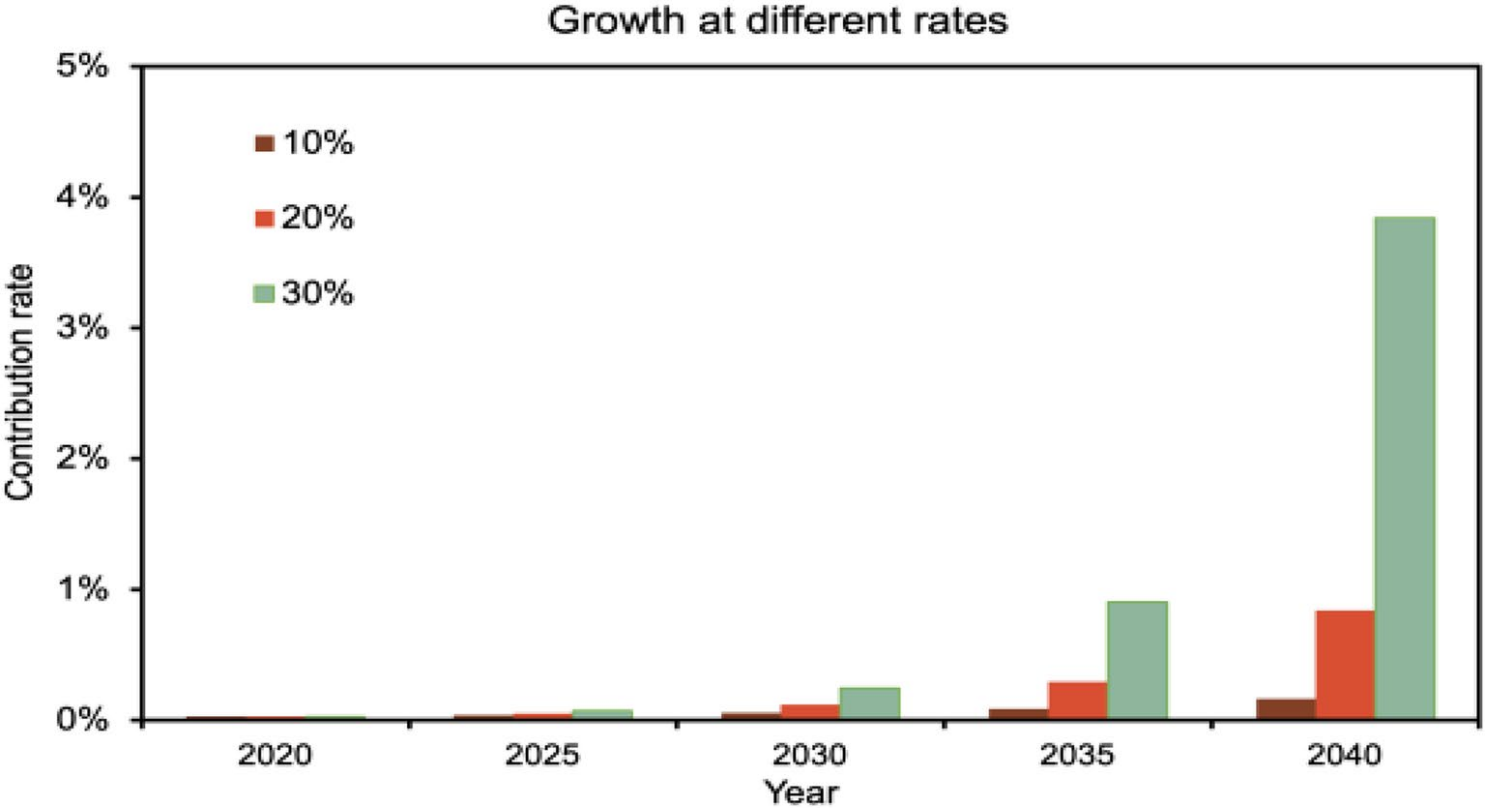
Differences under CCUS emissions reduction growth.

From Fig. 7. we can see that when CCUS emission reductions are set to grow at different rates, the difference in emission reduction contribution is more significant compared to the way the number of projects grows. When CO_2_ capture is increased at a 30% increase per year, the contribution can reach 3.8%, compared to an abatement contribution of about 0.016% when it is increased at a 10% rate. The difference between the two is about 20 times.

### Forecast CCUS sub-sector emission reduction potential

To further clarify the emission reduction contribution potential of CCUS technologies, the industrial sectors are subdivided ,the actual utilization of CCUS projects and industries are combined to compare the emission reduction contribution potential of CCUS technologies from an industry perspective.

At present, CCUS technology in China's industrial sector is mainly applied in the power industry, chemical industry and petroleum and cement industries, while there are no mature CCUS emission reduction projects of a certain scale in other industries. Therefore, we only predict the potential of CCUS to contribute to emission reductions in four industries: power industry, chemical industry, oil industry, and cement industry. The number of CCUS projects and captures in different industries by the end of 2020 are shown in Table 2.

**Table 2:** Sub-sector CCUS project information.

Sector	Carbon dioxide capture capacity (MT)	Number of CCUS projects
Chemical	2.05	13
Electricity	0.744	12
Petroleum	0.05	3
Cement	0.051	2

To set the growth rate of CCUS project emission reductions for the industry is ${\mathrm{k}}_{3}$,${\mathrm{k}}_{3}$ takes 10%, 20% and 30%, respectively. The industry emission reduction contribution is calculated as follows:

$${\mathrm{r}}_{\mathrm{i},\mathrm{t}}=\left[\sum {\mathrm{CCUS}}_{\mathrm{capi},\mathrm{t}-1}+{\mathrm{CCUS}}_{{\mathrm{cap}}_{\mathrm{i},2020}}\times {(1+{\mathrm{k}}_{3})}^{(\mathrm{t}-2020)}\right]/{\mathrm{emis}}_{\mathrm{i},\mathrm{t}}$$

where ${\mathrm{r}}_{\mathrm{i},\mathrm{t}}$ denotes the CCUS emission reduction contribution of industry $\mathrm{i}$ in year t, $\sum {\mathrm{ccus}}_{\mathrm{capi},\mathrm{t}-1}$ denotes the cumulative CCUS emission reduction of industry i in year t−1, ${\mathrm{ccus}}_{{\mathrm{cap}}_{\mathrm{i},2020}}$ denotes the CCUS emission reduction of industry i in 2020, and ${\mathrm{emis}}_{\mathrm{i},\mathrm{t}}$ is the CO_2_ emission of industry i in year t.

**Figure 8:**
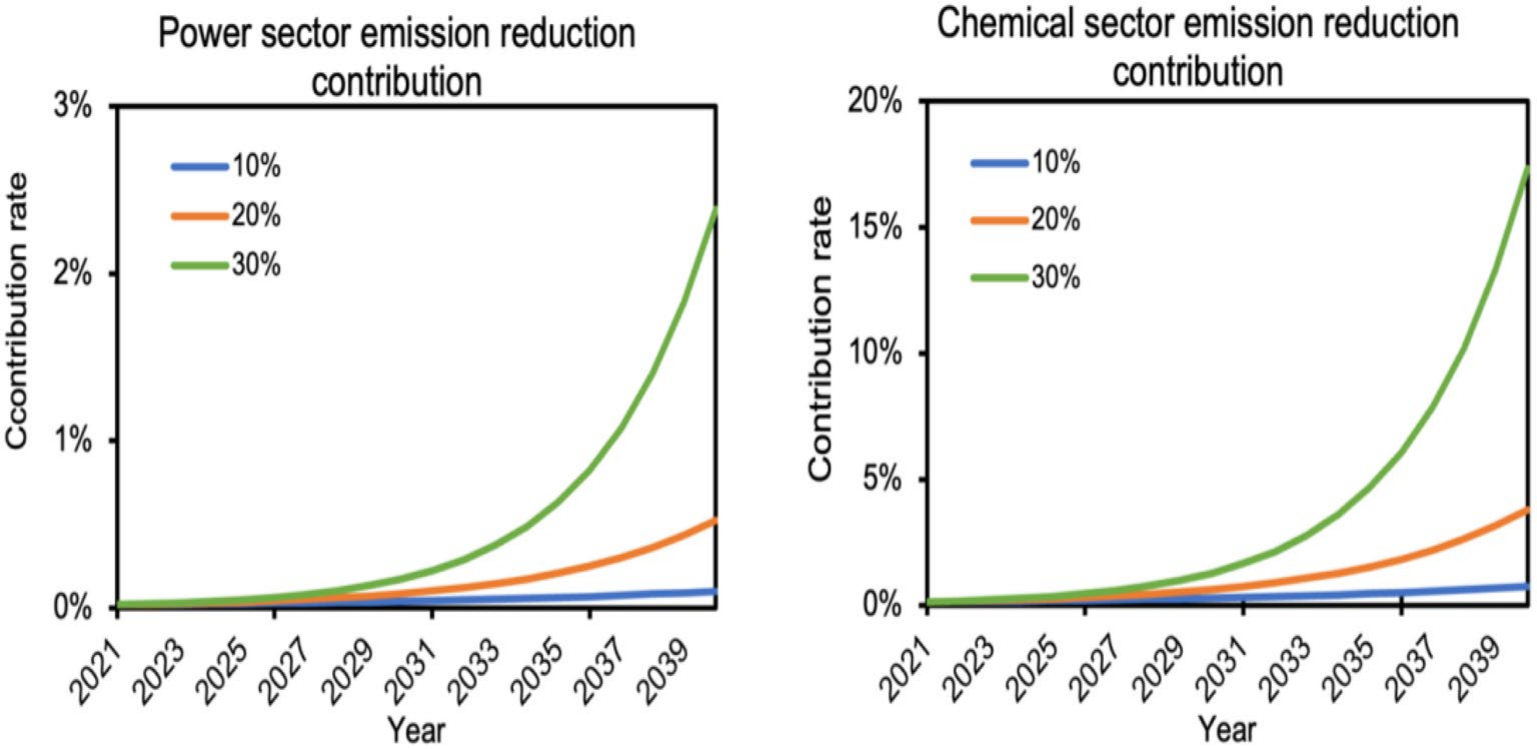
Potential for emission reduction contribution from the power and chemical industries.

It can be seen from Fig. 8**.** that the contribution of CCUS projects to emission reductions by 2040 varies greatly among industries due to the different current CCUS project emission reductions among industries, resulting in the same growth rate of capture volume. When the capture volume of the power sector grows at 30%, the contribution of CCUS technology to its emission reduction by 2040 only reaches 2.3%. In contrast, the projected emission reduction contribution of the chemical industry can reach 17.3% at a 30% growth rate, which is much higher than the emission reduction contribution of other industries.

### Forecast CCUS sub-regional emission reduction potential

The most important considerations for CCUS source-sink matching are the geographic location and environmental suitability of the emission source and the storage site^19^.The geological and geomorphological characteristics of China vary greatly from region to region, making the development potential of CCUS projects vary from region to region. Moreover, regional differences in economic development will also have an impact on CCUS emission reduction potential.

The regional emission reduction contribution is calculated by the following formula:

$${\mathrm{r}}_{\mathrm{j},\mathrm{t}}=\left[\sum {\mathrm{CCUS}}_{\mathrm{capj},\mathrm{t}-1}+{\mathrm{CCUS}}_{{\mathrm{cap}}_{\mathrm{j},2020}}\times {(1+{\mathrm{k}}_{3})}^{(\mathrm{t}-2020)}\right]/{\mathrm{emis}}_{\mathrm{j},\mathrm{t}}$$

where j represents the region, ${\mathrm{r}}_{\mathrm{j},\mathrm{t}}$ denotes the CCUS emission reduction contribution of region j in year t, $\sum {\mathrm{ccus}}_{\mathrm{capj},\mathrm{t}-1}$ denotes the cumulative CCUS emission reduction of region j in year t−1, ${\mathrm{ccus}}_{{\mathrm{cap}}_{\mathrm{j},2020}}$ denotes the CCUS emission reduction of region j in 2020, and ${\mathrm{emis}}_{\mathrm{j},\mathrm{t}}$ is the CO_2_ emission of region j in year t.

**Figure 9:**
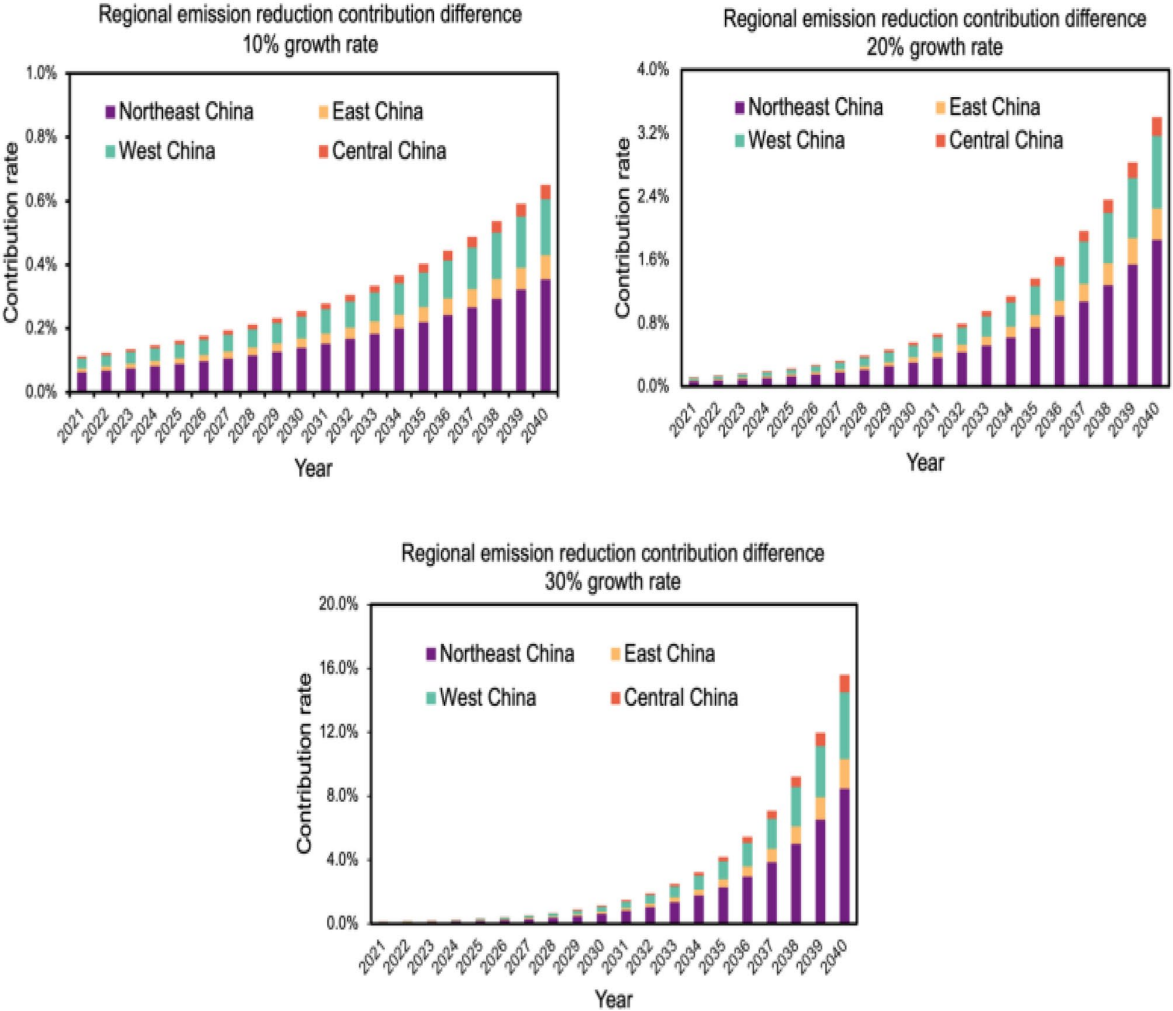
Differences in emission reduction contributions by region.

The contribution of CCUS technology which is shown in Fig. 9 varies significantly between regions at three different growth rates. The contribution is consistently the largest for the Northeast, where CCUS technology can contribute 8.4% to the Northeast's emissions reduction by 2040 when CO_2_ capture grows at 30%. Meanwhile, the contribution of CCUS emission reduction in the eastern region, which is the most developed region in China, is lower than that in the western region, accounting for about 40% of the CCUS emission reduction contribution in the western region. For the central region, the contribution is always the lowest at all three growth rates, and when the emission reduction scale is increased by the largest amount, the emission reduction contribution of CCUS technology is only 1.08%.The reason for this difference in contribution is that the Northeast region has lower carbon emissions compared to other regions, with only 1.641 billion tons of CO_2_ emissions projected by 2040, much lower than the Central region's 3.385 billion tons, the Eastern region's 6.181 billion tons, and the Western region's 4.606 billion tons. In addition, the Songliao Basin in northeast China has great geological potential for CO_2_ storage due to its good reservoir and cover properties. The large number of oil fields in the northeast also provides a way to utilize CO_2_, and the good geological conditions and source-sink match make the CCUS technology the most important contribution to the northeast. The eastern region has a better spatial distribution in terms of source-sink matching, with a large number of chemical companies and coal power plants with high CO_2_ concentration emission sources, but the lack of suitable geology for sequestration in the eastern region prevents the large-scale application of CCUS technology, making the predicted contribution lower than that of the western and northeastern regions.

## Evaluation of CCUS technology economics

As a key emission reduction technology, technical economics of CCUS technology is also of great concern in the process of promoting its utilization. Since the current investment cost of CCUS technology is too high, and the utilization of CO_2_ is mainly focused on EOR, industrial and food processing, and geological storage, which have multiple uncertainty effects, it is difficult for CCUS projects to have sustainable and stable income. In the absence of stable revenue, if policies and funds are heavily tilted to support CCUS technology, it is likely that satisfactory emission reduction results will not be achieved.

In order to evaluate the economics of CCUS in future development, we predict the annual cost of new CCUS technology abatement compared with our GDP (Gross National Product) in that year to obtain the ratio of CCUS technology abatement cost to domestic GDP as a measure of the economics of CCUS technology abatement.

**Figure 10:**
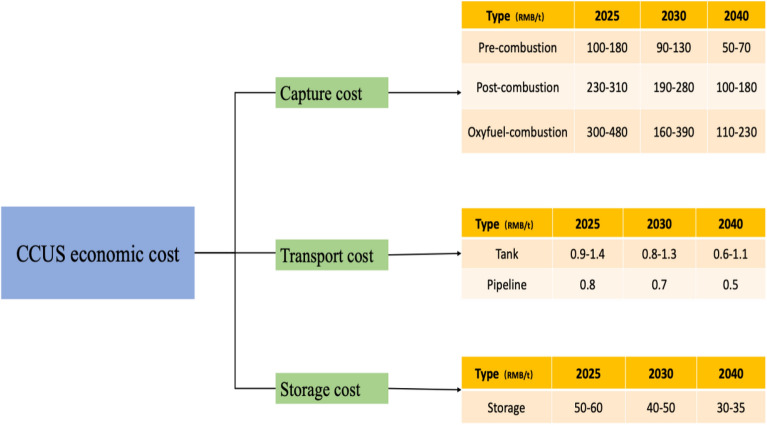
Diagram of CCUS cost (data from^22^).

The cost of CCUS technology includes economic cost and environmental cost, where economic cost includes fixed cost and operation cost which can be seen in Fig. 10, and environmental cost includes environmental risk and energy consumption emission. The cost per unit of CO_2_ reduction is obtained by combining them. The investment cost of CCUS technology will tend to decrease year by year due to the scale effect. In the early stage of CCUS utilization, new CCUS projects are constrained by technology and immaturity of operation management, so the cost is high in the early stage. With the breakthrough of technology bottleneck, the increasing maturity of operation management, some cluster projects share the infrastructure of pipeline, etc., the cost will keep decreasing.

The application of CCUS technology for emission reduction is mainly in the power industry. Compared to pre-combustion capture and oxygen-enriched combustion, post-combustion capture provides a solution for carbon reduction in coal-fired power plants and is currently the most widely used capture method. Therefore, when calculating the economics of CCUS, we make the important assumption that post-combustion capture is the primary capture method used to calculate costs.

The rate of CCUS development is influenced by external uncertainties, so we set different rates of CCUS development. The emission reductions of CCUS technology were obtained for annual growth of 10%, 20%, and 30%. The results are shown in the following Table 3**.**

**Table 3:** CCUS emission reductions at different growth rates.

Year	Reduction (MTPA)		
	10% growth rate	20% growth rate	30% growth rate
2025	422.83	598.86	824.84
2030	680.98	1490.14	3062.58
2035	1096.72	3707.96	11,371.14
2040	1766.27	7688.82	42,220.26

The growth rate of our GDP is 4.4% and 3.6% during 2021–2030 and 2031–2040, respectively, using the data measured by Goldman Sachs Group. According to the Goldman Sachs Group forecast growth rate, the National Bureau of Statistics 2020 China's gross national product as a starting value, GDP forecast values are shown in the following Table4.

**Table 4:** China's GDP forecast.

Year	GDP (trillion)
2025	1,260,057.3
2030	1,562,761.8
2035	1,865,054.7
2040	2,225,821.6

The cost projections for the CCUS sub-segments are summed to obtain a range of values for the unit cost of abatement of CCUS technology, using the following equation:

$${\mathrm{Cost}}_{\mathrm{t}}={\mathrm{uc}}_{\mathrm{t}}*{\mathrm{ccus}}_{{\mathrm{cap}}_{\mathrm{t}}}$$

where, ${\mathrm{Cost}}_{\mathrm{t}}$ represents the meaning of the total cost of CCUS investment in that year, ${uc}_{t}$ represents the unit cost, ${\mathrm{ccus}}_{{\mathrm{cap}}_{\mathrm{t}}}$ is the amount of emission reduction at year t, to get the total cost of CCUS emission reduction in that year, and with the GDP forecast value of that year to calculate. Based on the results of the above equation, the ratio of China's CCUS abatement investment to GDP is measured as Table 5.

**Table 5:** Percentage of CCUS investment in GDP.

Year	Percentage of CCUS investment in GDP
2025	0.00097–0.00243%
2030	0.00101–0.00649%
2035	0.00115–0.01592%
2040	0.00104–0.04099%

The data in the table reflects the CCUS in the continuous development process, due to the growing scale of the deployed projects, although the investment cost per unit of CO_2_ abatement is decreasing, the cost of investing in CCUS is increasing, from the initial less than 0.001% of the current year's GDP all the way up to about 0.04% of the current year's GDP in 2040. In 2040, CCUS will reduce about 2.44% of the national emissions, which is a capital-saving emission reduction technology with good overall emission reduction economics and a small investment in GDP.

## Conclusions

In this paper, we forecast the contribution of CCUS projects to the nation, industry, and region in 2040 under different development approaches.

The results of the study show that (1) when the number of new CCUS projects is 1, 2, and 3 per year nationwide, and the annual capture scale of the new projects is 1 million tons, the emission reduction contribution of CCUS technology is 0.22%, 0.32%, and 0.44%, respectively. When the amount of carbon dioxide captured by CCUS projects nationwide grows at different percentage rates, with growth rates of 10%, 20%, and 30%, the emission reduction contribution of CCUS projects is 0.51%, 2.08%, and 7.68%, respectively. (2) The types of CCUS project capture are divided into power industry, chemical industry, petroleum industry, and cement industry by industry. At present, the application of CCUS technology is mainly concentrated in the power and chemical industries. By 2040, the ease of reducing emissions using other technologies will be different for different industries due to the different carbon emissions in different industries. Therefore, the industry emission reduction contribution of CCUS technology in 2040 varies. The contribution of CCUS to industry emission reduction is 0.09%, 0.52%, and 2.38% when the annual CO_2_ capture scale of power industry grows at 10%, 20%, and 30%, respectively. When growing at the same rate, the contribution of CCUS to the industry's emission reduction in the chemical industry is 0.72%, 3.78%, and 17.31%, respectively. (3) When the country is divided into eastern, western, central, and northeastern regions according to the economy, the northeastern region has been the largest contributor to emission reduction by CCUS technology because the industry is less developed than other regions but has the Song Liao Basin and a large number of oil fields that can utilize CO_2_, and the regional contribution to emission reduction by 2040 is 0.35%, 1.85%, and 8.47%. The main reason behind this is that the central region lacks the geological conditions to promote the use of CCUS and cannot generate profit for the project. This paper still has some limitations in its contribution prediction for CCUS, first of all, the article ignores the source-sink matching problem to a certain extent, which makes the expansion of the CCUS program not fast. But in fact, when CCUS infrastructure is built in a region, it will make the sources of carbon dioxide emissions in the region share the CCUS infrastructure at a lower cost. This will accelerate the development of CCUS projects to a certain extent. Second, the iron and steel industry, which accounts for about 15% of China's CO2 emissions, was not considered. This is due to the fact that there are currently no mature CCUS projects in the steel industry. In the future, if CCUS projects in the iron and steel industry are successfully implemented, the contribution of the iron and steel industry to the reduction of emissions should also be predicted. Finally, this paper completely ignores the impact of policy. China's CCUS development will be further accelerated with the improvement of infrastructure if the government makes efforts to promote CCUS land construction, pipeline construction, and injection well construction. Incorporating policy factors as a consideration is also one of the directions for future research breakthroughs.

In order to predict the contribution of CCUS technology to national, industry and regional emission reduction, and in the light of the specific development situation in China, the following policy recommendations are made for the cause of emission reduction in China:

1. According to the contribution of CCUS to the emission reduction of different regions, it can be seen that the eastern region, as the most economically developed region in China, does not have the highest contribution to the emission reduction of CCUS. This is the direction that China should focus on in promoting the development of CCUS in the future. The eastern region has a very high density of industrial enterprises, and the carbon emissions are much higher than other regions in China, but due to the high population density in the eastern region, there is a lack of suitable land-based storage sites, which makes the development of CCUS slow. Therefore, the eastern region can conduct offshore geological exploration, consider using pipelines to transport CO_2_, and select suitable industrial clusters on land to jointly build infrastructure and reduce capital expenditures. After the initial formation of CCUS clusters in the eastern region, the experience of its capital operation can be extended to the western region, northeastern region and other regions with storage locations to further develop CCUS.
2. Further improve the carbon trading market and subsidize CCUS enterprises: The high cost and unstable income of CCUS make enterprises reluctant to use CCUS to reduce emissions. The state can further improve the carbon trading market, so that the carbon credits of CCUS emission reduction can be traded again in the market to obtain income and reduce the burden of enterprises. In addition, the government can also support the development of CCUS by providing financial policies such as land premium exemptions and tax exemptions for CCUS projects in the process of construction.
3. Putting CCUS to use in multiple industries. When the amount of CO_2_ captured by CCUS technology increases by 30%, the contribution to China's emission reduction is still only 7.68%. One of the reasons for the low contribution rate is that there is no single industry where CCUS can be applied, for example, there is no large-scale utilization of CCUS technology in China's iron and steel industry, which accounts for nearly 15% of the country's carbon emissions. Therefore, we can try to organize capital and technology operation in the iron and steel industry led by the state and build a demonstration project of emission reduction in the iron and steel industry, so as to create a case for other enterprises to learn from.
4. Combine CCUS with other clean energy sources to promote carbon neutrality. According to the development speed and prediction results in the paper, it can be concluded that the contribution of CCUS technology to China's carbon emission reduction in 2040 is not outstanding, and there is still a certain gap between it and the realization of the goal of carbon neutrality. In order to make up for this gap, in addition to accelerating the development of CCUS, broadening the application industries, and lowering the cost of the technology, we should also strengthen the utilization of new energy technologies, and combine new energy technologies with CCUS technologies to reduce the impact on the economy during the process of carbon neutrality. The proposed Blue Hydrogen Project in Tangshan City provides a good blueprint for the combination of technologies. The Caofeidian District of Tangshan City is initially planning to form a cluster of new energy industries such as photovoltaic power generation, offshore wind power, gas-steam combined cycle power generation, hydrogen production, and CCUS, etc., and the economies of scale, comparative advantages, and knowledge spillover effects of the industrial clusters will bring the full potential of CCUS to bear on emissions reduction.

## Data Availability

The datasets used or analysed during the current study available from the corresponding author on reasonable request.The datasets used or analysed during the current study available from the corresponding author on reasonable request.

## Abbreviations

CCS: Carbon capture and storage
CCU: Carbon capture and use
CCUS: Carbon capture, utilization and storage
IEA: International Energy Agency
GDP: Gross domestic product
PC: Pulverized coal
$${\text{CO}}_{2}$$: Carbon dioxide
${\text{CO}}_{2}$-EOR: Carbon dioxide enhanced oil recovery
${\text{CO}}_{2}$-ECBM: Carbon dioxide enhanced coal bed methane recovery
T: Tonne
MT: $${10}^{6}$$ Tonnes
MTPA: ${10}^{6}$ Tonnes per annum
IGCC: Integrated gasification combined cycle
NGCC: Natural gas combined cycle
