# Author Correction: CCUS development in China and forecast its contribution to emission reduction

**DOI:** 10.1038/s41598-023-49301-z

**Published:** 2023-12-19

**Authors:** Pengchen Wang, Beibei Shi, Nan Li, Rong Kang, Yan Li, Guiwen Wang, Long Yang

**Affiliations:** 1 School of Economics and Management, Northwest University, Xuefu Avenue No.1, Chang’an District, Xi’an, 710127 China; 2 China Energy JinJie Energy Co., Ltd, JinJie Industrial Park, Shenmu, 719319 Yulin China

Correction to: *Scientific Reports* 10.1038/s41598-023-44893-y, published online 19 October 2023

The original version of this Article contained an error in Figure 1 and 5 where the figures were incomplete. The original Figure 1 and Figure 5 accompanying legend appear below.

**Figure 1:**
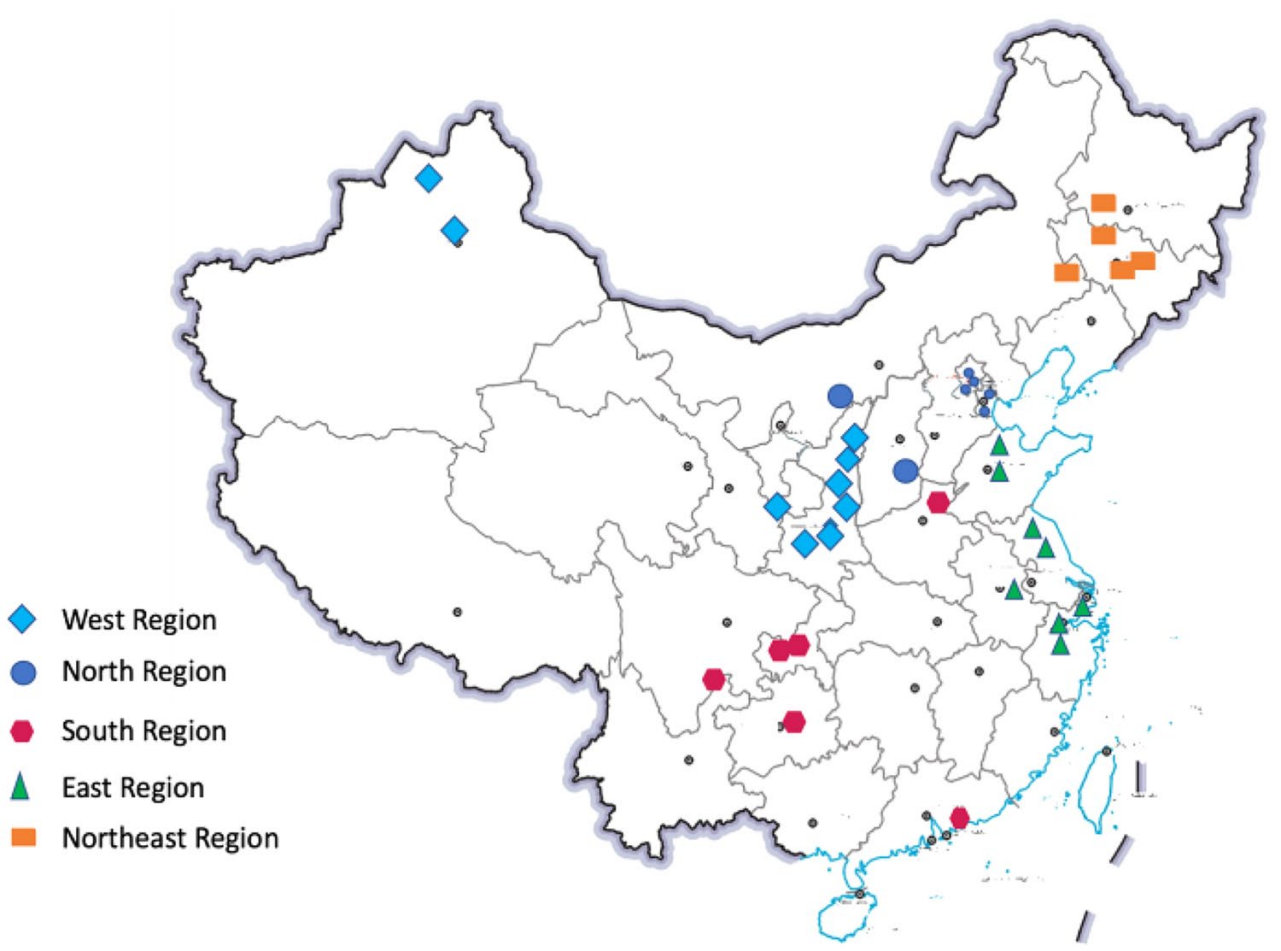
CCUS information location distribution map25,26.

**Figure 5:**
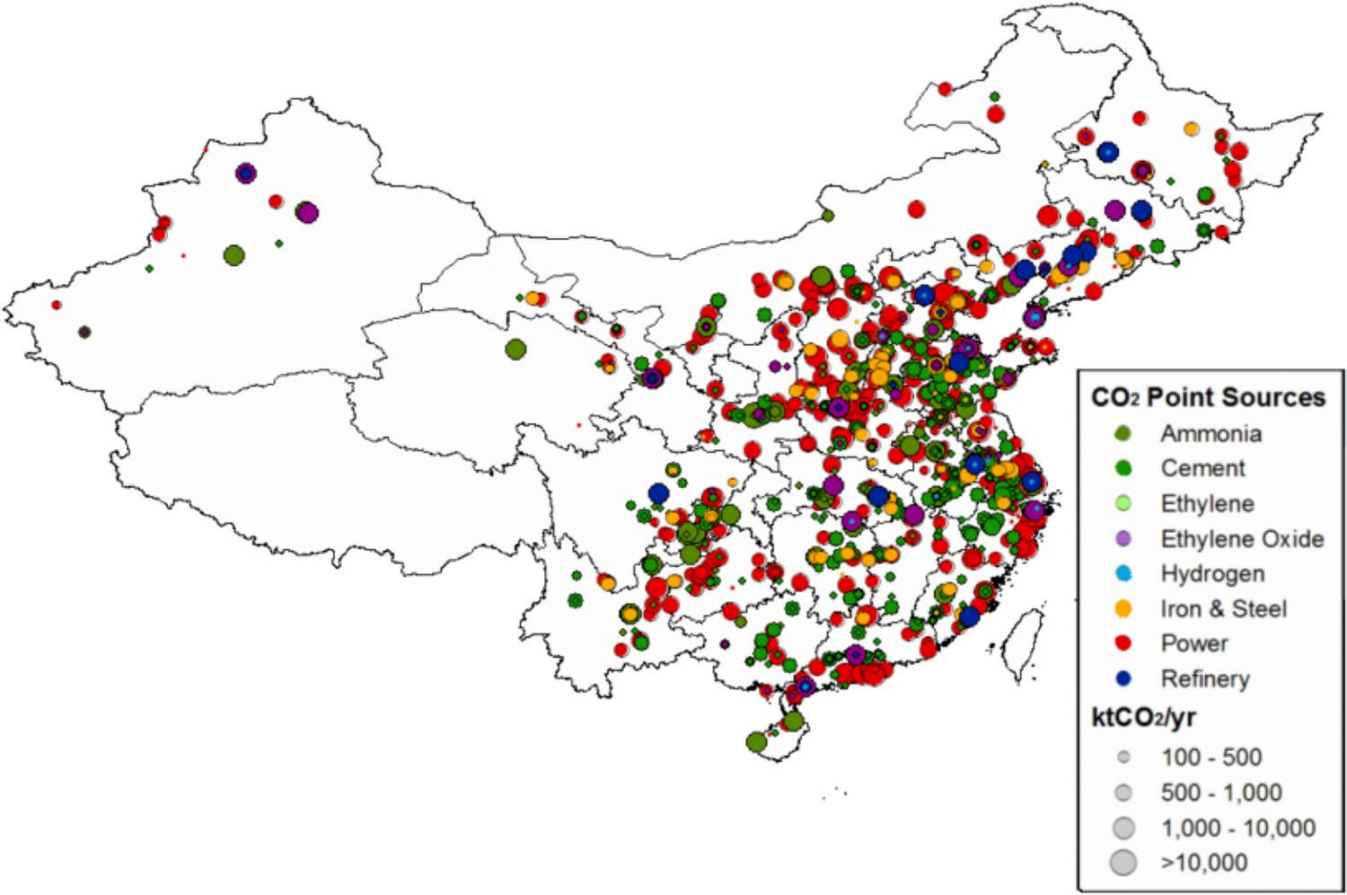
Distribution of large CO_2_ point sources in China29.

The original Article has been corrected.

